# The molecular diversity of transcriptional factor TfoX is a determinant in natural transformation in *Glaesserella parasuis*

**DOI:** 10.3389/fmicb.2022.948633

**Published:** 2022-07-29

**Authors:** Xiaoyu Tang, Zhen Yang, Ke Dai, Geyan Liu, Yung-Fu Chang, Xinwei Tang, Kang Wang, Yiwen Zhang, Bangdi Hu, Sanjie Cao, Xiaobo Huang, Qigui Yan, Rui Wu, Qin Zhao, Senyan Du, Yifei Lang, Xinfeng Han, Yong Huang, Xintian Wen, Yiping Wen

**Affiliations:** ^1^Research Center of Swine Disease, College of Veterinary Medicine, Sichuan Agricultural University, Chengdu, China; ^2^Department of Population Medicine and Diagnostic Sciences, College of Veterinary Medicine, Cornell University, Ithaca, NY, United States

**Keywords:** *Glaesserella parasuis*, natural transformation, *tfoX*, molecular diversity, competence gene

## Abstract

Natural transformation is a mechanism by which a particular bacterial species takes up foreign DNA and integrates it into its genome. The swine pathogen *Glaesserella parasuis* (*G. parasuis*) is a naturally transformable bacterium. The regulation of competence, however, is not fully understood. In this study, the natural transformability of 99 strains was investigated. Only 44% of the strains were transformable under laboratory conditions. Through a high-resolution melting curve and phylogenetic analysis, we found that genetic differences in the core regulator of natural transformation, the *tfoX* gene, leads to two distinct natural transformation phenotypes. In the absence of the *tfoX* gene, the highly transformable strain SC1401 lost its natural transformability. In addition, when the SC1401 *tfoX* gene was replaced by the *tfoX* of SH0165, which has no natural transformability, competence was also lost. These results suggest that TfoX is a core regulator of natural transformation in *G. parasuis*, and that differences in *tfoX* can be used as a molecular indicator of natural transformability. Transcriptomic and proteomic analyses of the SC1401 wildtype strain, and a *tfoX* gene deletion strain showed that differential gene expression and protein synthesis is mainly centered on pathways related to glucose metabolism. The results suggest that *tfoX* may mediate natural transformation by regulating the metabolism of carbon sources. Our study provides evidence that *tfoX* plays an important role in the natural transformation of *G. parasuis*.

## Introduction

Natural transformation, a horizontal gene transfer (HGT) mode of bacteria, allows bacteria to actively uptake foreign DNA under natural conditions and integrate it into the genome through homologous recombination (Lorenz and Wackernagel, 1994). Natural transformation plays a vital role in the rapid spread of pathogenic virulence factors and resistance genes, resulting in the emergence of multidrug-resistant or highly pathogenic strains (Domingues, Harms et al., 2012; Domingues, Nielsen et al., 2012; Domingues et al., 2019). Unlike electroporation or chemical transformation (Mell et al., 2012), natural transformation is an active process that relies on proteins encoded by the recipient cell. Competence is the physiological state in which the natural transformation of bacteria can occur (Fontaine et al., 2015). The primary process of natural transformation can be divided into the uptake of foreign genes and their integration into the bacterial DNA (Chen and Dubnau, 2004). The transformation process requires the precise coordination of multiple competence genes, which are highly conserved in transformable bacteria. The encoded proteins perform the functions of DNA uptake, transport, and recombination processing that allow foreign DNA to enter and integrate into the genome of the recipient bacteria (Chen and Dubnau, 2004; Dubnau and Blokesch, 2019). For gram-negative bacteria, the type IV pilus system plays a vital role in the uptake of exogenous DNA (Costa et al., 2021). Double-stranded DNA (dsDNA) is captured by the extracellular fimbriae protein PilA, and through the contraction of the pilus, the dsDNA passes through the outer membrane through the activity of the secretory porin PilQ. Then the dsDNA binds to the DNA-binding protein ComEA in the periplasm of the cell and delivers the dsDNA to the inner membrane channel protein ComEC. The dsDNA is then unwound into single-stranded DNA (ssDNA); one strand enters the cytoplasm through ComEC, and the other is degraded. The ssDNA entering the cytoplasm can be bound to an ssDNA-binding protein encoded by *ssb*, protecting it from endogenous nuclease degradation. SsB presents the ssDNA to DprA, a DNA-processing protein. Subsequently, the ssDNA and genome integration are promoted through the synergistic action of RecA and a DNA helicase encoded by *comM* (Antonova and Hammer, 2015; Hepp and Maier, 2016).

Due to different growth environments, bacteria have evolved in various ways to induce competence, and nutritional conditions are the critical factors of many pathogenic bacteria (Seitz and Blokesch, 2013). As an essential element of microbial growth, the carbon source is involved in a variety of metabolic processes and regulatory pathways, and its effects on natural transformation have also been reported in many bacteria. For example, the natural transformation frequency of *E. coli* could be increased by adding a common carbon source in the intestinal culture medium (Guo et al., 2015); the formation of competence of *Haemophilus influenzae* was influenced by the PTS carbon source (Macfadyen and Redfield, 1996).

Although competence genes are well-conserved in bacteria, it has been commonly reported that the transformability of different isolates of competent species is variable. For instance, nearly 60% of *H. influenzae* and *Pseudomonas stutzeri* isolates are non-transformable, and the transformation frequency was also significantly different (Maughan and Redfield, 2009; Durieux et al., 2019). Comparable variation has also been found in *Glaesserella parasuis* (*G. parasuis*), a naturally transformable, gram-negative, pleomorphic bacterium of the *Pasteurellaceae* group (Bigas et al., 2005; Dai et al., 2021). Although a comparison of the entire genome sequence of *G. parasuis* isolates published in NCBI showed that all strains contained the identified homologous genes required for natural transformation, such as *pilA*, *comEA*, *comM*, etc., many *G. parasuis* isolates are non-transformable (Dai et al., 2016, 2018b). The molecular mechanism of such diversity is unclear, however, it may due to significant differences in the regulatory systems of the different strains. TfoX (encoded by *tfoX*, the orthologous gene is *sxy*) is a core regulatory factor of natural transformation in a variety of Gram-negative bacteria (Metzger et al., 2019; Sondberg et al., 2019). TfoX-deficient mutant strains of *H. influenzae*, *Vibrio cholerae* and *Vibrio fischeri* have lost their intrinsic natural transformability (Sinha et al., 2013; Lo Scrudato et al., 2014). The regulatory pathways of TfoX are diverse in different bacteria, but they all ultimately act on downstream competence genes. TfoX interacts synergically with the cAMP-CRP complex to activate transcription of the competence genes containing noncanonical CRP binding sites (CRP-S), thus regulating the natural transformation of bacteria (Sinha et al., 2012). In our previous studies, we analyzed the biologic functions of a homolog *tfoX* in SC1401, a *G. parasuis* strain with a high natural transformation efficiency (Dai et al., 2016). We found that other genes (e.g., *comABCDEF*, *pilABCD*, *cyaA*, *crp*, etc.) associated with natural transformation were highly conserved with 90% nucleotide sequence identity, while the DNA sequence of *tfoX* from *G. parasuis* SC1401 to that of strain SH0165 had only 74% nucleotide sequence identity. A proline residue was found inserted into *G. parasuis* SC1401 TfoX at codon 64th. Moreover, a secondary structure comparison showed that SC1401TfoX harbors two more α-helices (Dai et al., 2018a). Interestingly, SH0165 is a non-transformable strain (Zhang et al., 2015). The heterogenicity of *tfoX* among *G. parasuis* strains suggests that *G. parasuis* is unique in its regulation of natural transformation, but the molecular mechanism is still to be determined.

The mechanism of the regulation of natural transformation in *G. parasuis* occurs largely *via* the product of *tfoX*, a gene shown to have significant heterogenicity among *G. parasuis* strains. In this study, we analyzed the *tfoX* genes from 99 strains of *G. parasuis* using a High-Resolution Melting (HRM) analysis and gene sequencing to measure the nucleotide sequence identity of this locus. We then measured the natural transformation frequency of these strains and found that sequence variations led to two distinct natural transformation phenotypes, suggesting that differences in *tfoX* could play an important role in determining the natural transformability of various *G. parasuis* strains.

## Materials and methods

### Strains, primer, plasmids, and growth conditions

The bacterial strains and plasmids used in this study are listed in Supplementary Table S1, and primers are listed in Supplementary Table S2. *Escherichia coli* (*E. coli*) DH5α, used to propagate the plasmids, was cultured in Luria-Bertani (LB, Difco, United States) broth with shaking or LB agar (Invitrogen, China) plates supplemented with 50 μg ml^−1^ kanamycin (Sigma-Aldrich, United States) when required. *G. parasuis* was cultured in Tryptic Soy Broth (TSB, Difco, United States) with shaking or Tryptic Soy agar (TSA, Difco, United States) supplemented with 5% inactivated bovine serum (Solarbio, China) and 0.1% (w/v) nicotinamide adenine dinucleotide (NAD, Sigma-Aldrich, United States; TSB++ and TSA++). When necessary, these media were supplemented with 50 μg ml^−1^ of kanamycin or 100 μg ml^−1^ erythromycin. All strains were grown at 37°C. Serotypes of *G. parasuis* were identified using PCR-based molecular serotyping (Howell et al., 2015).

### Natural transformation methodology

The specific protocol for natural transformation was performed as previously described (Dai et al., 2021). Briefly, the recipient bacteria were isolated into 3 ml TSB++ and shaken at 37°C for 14 h to an optical density 600 (OD600) = 1.8; late-stationary phase. The bacteria were then spotted on TSA++, cultured for 13 h at 37°C, and resuspended in 30 μl TSB++ adjusted to a density of 5 × 10^10^ cfu/ml. A 20 μl aliquot was extracted and supplemented with 1 μl of cAMP (final concentration of 8 mM) and 2 μg of donor DNA [genomic DNA of SC1401Δ*htrA*::Kan (Zhang et al., 2016), SC1401Δ*cdt*::Erm or a recombinant plasmid constructed in this study]. The mixture was incubated for 10 min at 37°C and then spotted on TSA++ again, and incubated for 5 h at 37°C to induce the expression of antibiotic resistance. Afterwards, bacteria were harvested and resuspended in 100 μl of TSB++ and plated on a selective medium after serial dilution. The diluted bacteria (about 10^−7^) were plated on TSA++. Bacteria were incubated for 1–2 days. The formula of natural transformation frequencies (NTF) is kanamycin/erythromycin-resistant colony-forming units (CFU) ml/total CFU as previously described (Humbert et al., 2011).

### High-resolution melting (HRM) analyses

DNA samples were isolated from *G. parasuis* clinical isolates using the TIANamp Bacteria DNA Kit (Tiangen, China) according to the manufacturer’s instructions. The HRM primers (P1, P2) were designed to amplify the sequences (approximately 100 bp) surrounding the target site. The annealing temperatures of the HRM primers were optimized to approximately 60°C and purified by HPLC.

A sample of 20 ng of the genomic DNA of a *G. parasuis* strain was used as a template in a 20 μl PCR reaction using a LightCycler® 480 High Resolution Melting Master kit (Roche), with 1× LightCycler® 480 High Resolution Melting Master Mix, and final concentrations of 200 nM of forward and reverse primers and 3 mM MgCl_2_. A total of 108 bp PCR products surrounding the target site were amplified in the LightCycler® 96 instrument (Roche), using the following cycling conditions: pre-incubation 10 min 95°C, 45 cycles 3-step amplification (95°C for 10 s, 57°C for 10 s, 72°C for 20 s) followed by one cycle of high resolution melting [95°C for 60 s, 40°C for 60 s, 65°C for 1 s, and finally using a ramp rate 0.07°C/s and an acquisition mode setting of 15 readings/s to reach the target temperature 95°C (1 s)]. Raw data were analyzed using the LightCycler® 96 software 1.1 (Roche), which sorted the samples into groups based on their normalized melting curves. Each sample included 2–3 technical replicates.

### Phylogenetic analysis of *tfoX* gene in *Glaesserella parasuis*

PCR fragments containing the *tfoX* gene were amplified from the genomic DNA of *G. parasuis* using the primers P3 and P4. The PCR products were submitted to Sangon Biotech for sequencing. Phylogenetic trees were made by MEGA v6 and visualized using the online server iTOL v4^1^ (Letunic and Bork, 2019). Neighbor-joining (NJ) method was utilized to analyze sequence distances among different *G.parasuis* strains. In all analyses, the Phylogeny reconstruction used maximum likelihood, and the phylogeny was tested with the bootstrap method, using 500 bootstrap replications.

### Construction of *tfoX* deficient mutant and replaced strains

To construct an in-frame nonpolar *tfoX* mutant, the target vector pK18-*tfoX* was built as follows: first, the 653-bp upstream homologous region and the 656-bp downstream homologous region of the *in-situ tfoX* gene were amplified using the primers P5/P6 and P7/P8 from the genomic DNA of *G. parasuis* SC1401, then the 935-bp kanamycin-resistant cassette (Kan^R^, *aminoglycoside phosphotransferase Aph* (*3′*)*-IIa* gene) was amplified by PCR from pKD4 using the primers P9/P10. Subsequently, these three fragments were purified using a Qiaquick spin column kit (Qiagen, Hilden, Germany) and were ligated into the linearized vector pK18 mobSacB. Similarly, the *tfoX* gene of SC1401 and SH0165 were amplified, and the four fragments were ligated into pK18 mobSacB to construct the target vector pK18-SC1401*tfoX*-Kan and pK18-SH0165*tfoX*-Kan. Finally, the resulting plasmids were transformed into the *G. parasuis* strain SC1401 using a natural transformation technique. Transformant bacteria were incubated for 36 h. The resultant kanamycin-resistant transformants were cultured on a large scale in TSB++/Kan for further identification by PCR.

### Quantitative real-time PCR

RNA samples were isolated from the *G. parasuis* strains or SC1401 derivatives (cultured in TSB++ to obtain a bacterial culture volume equivalent to 1 ml of OD_600_ = 1.8 or on TSA++ for 13 h, respectively) using the UNIQ-10 Column Total RNA Purification Kit (Sangon, China) according to the manufacturer’s instructions. Two-step RT-PCR was performed using the PrimeScriptTM RT reagent Kit with the gDNA Eraser (Takara, Japan). The competence gene transcripts were then analyzed using quantitative reverse transcription PCR (qRT-PCR) with the reagent of SYBR Premix EX TaqTM II (Tli RNaseH Plus; Takara, Japan). The 2 [–ΔΔC(T)] method was used to relatively quantitate the expression of multiple genes compared to the stably expressed 16S RNA reference gene. Runs of qPCR were performed with a Lightcycler96 (Roche, Switzerland) system. The experiments were performed in independent triplicates.

### Transcriptome sequencing and data analysis

Strains SC1401 and SC1401Δ*tfoX*::Kan were cultivated with three replicates in TSB++ at 220 rpm and 37°C. According to the manufacturer’s instructions, when the cultures had grown to the late-stationary phase, the total RNA of each sample was extracted using the Trizol reagent (Invitrogen, Carlsbad, CA, United States). After dissolving the RNA samples in RNase-free water, the total RNA degradation and contamination levels were monitored on 1% agarose gels. RNA quantification, library preparation, clustering, and sequencing were performed at Novogene Bioinformatics Technology Co., Ltd. (Beijing, PR China). The clean data (clean reads) were acquired by removing reads containing adapter sequence, reads containing poly-N, and low-quality reads from raw data. The *G. parasuis* SC1401 genome (CP015099.1) and the gene model annotation files were downloaded^2^ and used to build the index and to align clean reads of the *G. parasuis* SC1401 genome (CP015099.1) with Bowtie2-2.2.3 (Langmead et al., 2009).

To quantify the gene expression, the read numbers mapped to each gene were counted using the HTSeq v0.6.1 (Anders et al., 2015). The FPKM (expected number of Fragments Per Kilobase of transcript sequence per Million base pairs sequenced) of each gene was used to analyze the gene expression variations. In our study, differential expression analysis of *G. parasuis* SC1401 and *G. parasuis* SC1401Δ*tfoX* was performed using the DEGSeq R package (version 1.20.0; Anders and Huber, 2010). The resulting *p*-values were adjusted using the Benjamini and Hochberg approach to control the false discovery rate. Genes with an adjusted *p*-value <0.05 were considered to be significantly differentially expressed. The GOseq R package implemented a Gene Ontology (GO) enrichment analysis of differentially expressed genes to correct for gene length bias (Young et al., 2010). GO terms with a corrected *p*-value less than 0.05 were considered significantly enriched by differentially expressed genes. We used the KOBAS software (Center for Bioinformatics, Peking University & Institute of Computing Technology, Chinese Academy of Sciences) to test the statistical enrichment of differential expression genes in KEGG (Kyoto Encyclopedia of Genes and Genomes) pathways (Kanehisa et al., 2008; Young et al., 2010).

### Proteome sequencing and data analysis

Three replicates of the strains SC1401 and SC1401Δ*tfoX*::Kan were cultivated with in TSB++ at 220 rpm and 37°C. When the cultures had grown to the late-stationary phase, the total protein of each sample was extracted as previously described (Wisniewski et al., 2009). Protein concentrations were determined using the Bradford protein assay (Bio-Rad, United States). In addition, 10 μg of the total extracted proteins were analyzed by SDS-PAGE. Peptide preparation, TMT labeling, HPLC fractionation, and LC–MS/MS analysis of protein samples were performed at Novogene Bioinformatics Technology Co., Ltd. (Beijing, PR China).

The resulting spectra from each fraction were searched separately against the database^3^ using Proteome Discoverer 2.2 (PD 2.2, Thermo, United States). The confidence levels for the identified proteins were determined by the false discovery rate (FDR). Proteins containing similar peptides that could not be distinguished based on MS/MS analysis were grouped separately. Reporter Quantification (TMT) was used for TMT quantification. The protein quantitation results were statistically analyzed by the Mann–Whitney test, and the significance ratios, defined as *p* < 0.05 and |log2FC| > 1.2 were used to screen the differentially expressed proteins (DEP) as previously described.

To deepen our understanding of the potential biological functions of our *tfoX* genes, GO and KEGG enrichment analyses on the differentially expressed proteins were performed using hypergeometric tests to discover the biological processes and pathways involved. More specifically, biological process terms from the GO and KEGG pathways were chosen from the annotation resources provided to functionally characterize the driver genes identified.

### Statistical analysis

Transformation frequencies were determined from the number of antibiotic-resistant cfu mL^−1^ divided by the total cfu mL^−1^ scored on non-selective agar. Statistical analyses were performed using the GraphPad Prism 6 (GraphPad software, CA, United States). Statistical significance was evaluated using Student’s *t*-test, a one-way ANOVA, or a two-way ANOVA. The value of *p* < 0.05 represented statistically significant differences. Error bars in all figures represent the standard deviations of three independent experiments.

## Results

### The natural transformation frequency is variable in *G. parasuis* wild strains

**Figure 1:**
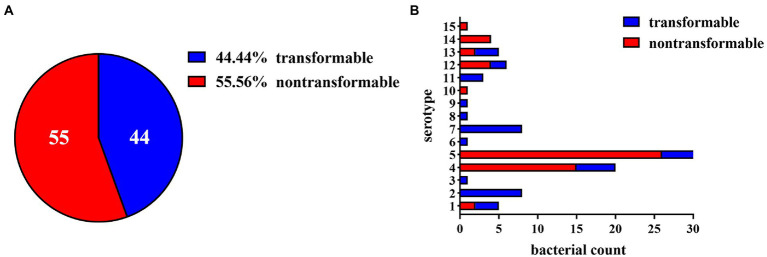
Distribution of transformable strains in *Glaesserella parasuis*. **(A)** Pie chart of the proportion of transformable and non-transformable isolates in 99 *G. parasuis* strains. **(B)** The distribution of transformable and non-transformable strains of different serotypes. The horizontal axis represents the number of bacteria, and the vertical axis represents the serotype. Regions in blue or red depict transformable or non-transformable strains, respectively. When the natural transformation frequency is greater than 1 × 10^−8^, we considered the strain to be transformable.

Despite the genes required for natural transformation in all sequenced *G. parasuis* strains and the nucleotide sequence homology of these genes exceeding 95%, many strains still appeared non-transformable under laboratory conditions. To gain insight into these extensive variations, we evaluated the natural competence and transformation ability of 15 standard strains and 84 clinical isolates. As shown in Figure 1A, 44 competent strains, and 55 strains with no natural transformability were identified under laboratory conditions. Furthermore, these transformable strains showed varying levels of natural transformation; some strains had high transformation frequencies (about 1.0 × 10^−4^), while others had low transformation frequencies (about 1.0 × 10^−7^). In brief, like many other competent bacteria, the natural transformability of *G. parasuis* varied greatly under the same experimental conditions.

### More transformable strains were distributed in low and medium virulence isolates

To explore whether the natural transformability of *G. parasuis* was related to bacterial virulence, we analyzed the serotypes of the 84 clinical isolates mentioned above. As shown in Figure 1B, serotypes 4 and 5 comprise the largest proportion of all of the strains investigated because these two serotypes are the most frequent in China. Most strains of these two serotypes have no transformability. In addition, serotypes 2 and 7 were abundant, and all isolates were found to have transformability. The remaining serotypes are relatively infrequent because they are rarely present in China. The results indicated that all of the low virulence strains (serotypes 3, 6, 7, 8, 9, 11) were naturally transformable. In contrast, only 45% of the moderate virulence strains (serotypes 2, 4, 15) and 29% of the high virulence strains (serotypes 1, 5, 10, 12, 13, 14) had natural transformability. This result demonstrates that the virulence of *G. parasuis* does not completely determine its natural transformability status. However, there were significantly more naturally transformable strains in the medium and low virulence strain groups, suggesting that the virulence factors of *G. parasuis* may be related to the natural transformability of these bacteria.

### Two molecular types of the *tfoX* gene are present in *Glaesserella parasuis*

**Figure 2:**
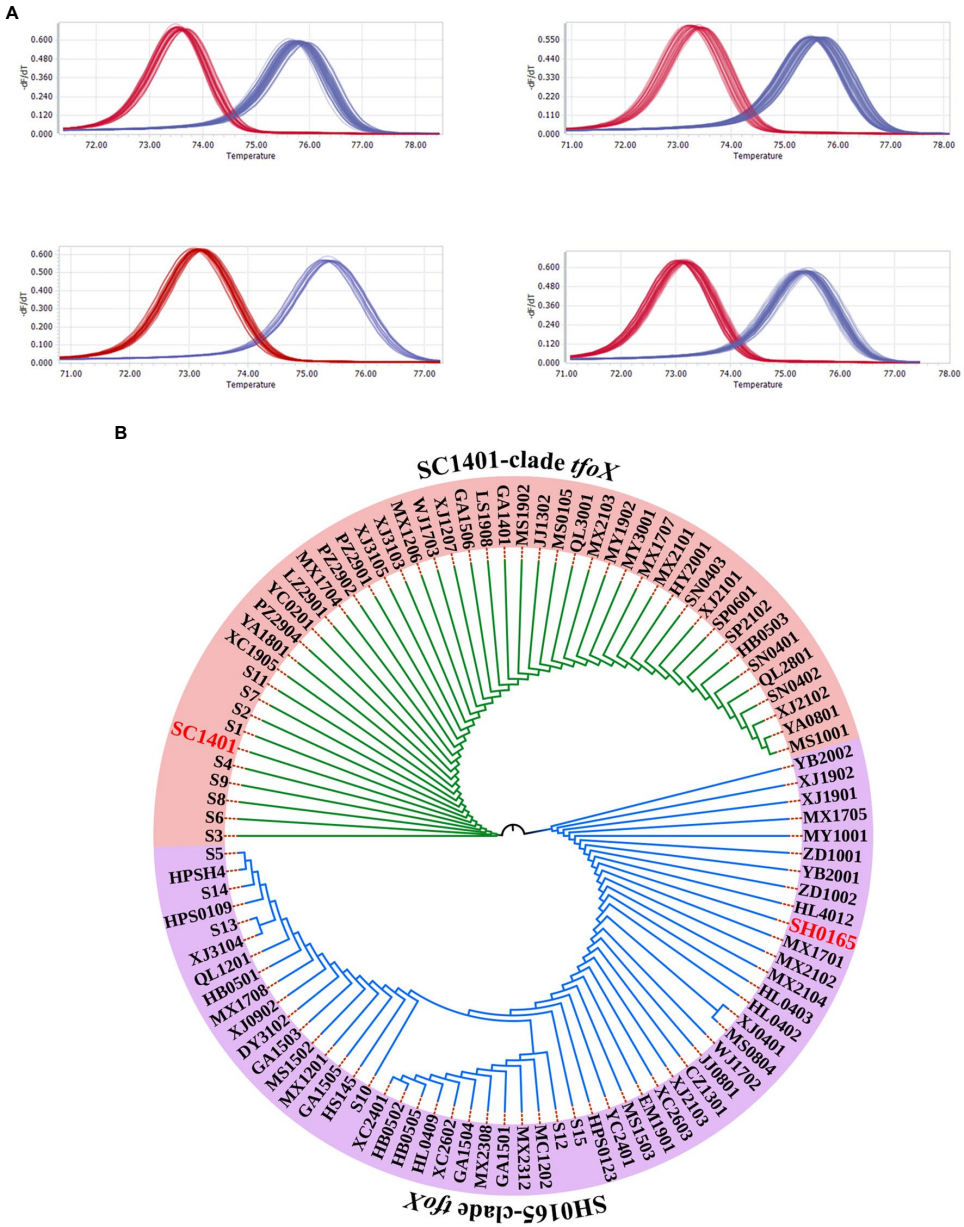
*tfoX* molecular differences in *Glaesserella parasuis*. **(A)** High resolution melting curves of the *tfoX* genes of 99 *G. parasuis* strains. A HRM analysis showed that two types of melting curves occurred: the red and blue curves are consistent with SC1401*tfoX*, or SH0165*tfoX*, respectively. **(B)** Phylogenetic tree of *tfoX* in *G. parasuis*. A neighbor-joining (NJ) tree demonstrated the overall diversity of *tfoX* from *G. parasuis.* The results are consistent with the HRM analysis, and all *tfoX* genes are evolutionarily divided into two clades.

In our previous study, we sequenced the highly transformable strain SC1401 and compared it to the *G. parasuis* reference strain SH0165, which is non-transformable under all of the experimental conditions we tried. Intriguingly, we found that the nucleotide sequence identity of *tfoX* gene between the two strains was 74%, and the amino acid sequence identity was 73%. Further comparison with *tfoX* genes of the sequenced strains showed that sequence differences are widely found in *G. parasuis* strains. In contrast, *tfoX* is conserved in *E. coli* and in *H. influenzae* (Dai et al., 2018a). To explore the distribution of the *tfoX* gene variants in *G. parasuis*, the 99 *G. parasuis* isolates referred to in Section “The Natural Transformation Frequency Is Variable in *G. parasuis* Wild Strains” were genotyped using a high-resolution melting (HRM) analysis. As shown in Figure 2A, the *tfoX* gene of all tested strains could be divided into two categories, one with a melting peak Tm value between 72°C and 74°C, similar to SC1401, and another with a melting peak Tm value between 75°C and 77°C, similar to SH0165.

We next sequenced the *tfoX* gene of each *G. parasuis* strain, and compared and analyzed the sequence homology *via* a phylogenetic analysis. The results showed that *tfoX* evolved into two clades, SC1401 and SH0165, consistent with the HRM results (Figure 2B). According to *tfoX* gene variants, we divided the 99 *G. parasuis* strains into SC1401 *tfoX* strains and SH0165 *tfoX* strains, and these strains represented two different genotypes of *G. parasuis*. Since the *tfoX* gene has been identified as a core regulator in other bacterial species, we speculated that this molecular level difference could be a key determinant of natural competence.

### *tfoX* gene variants lead to different natural transformation phenotypes of *Glaesserella parasuis*

The *tfoX* typing and natural transformability of all strains were assessed to determine the relationship between the *tfoX* gene sequence and the natural transformation phenotype. It is worth mentioning that the natural transformation capacity was consistent with the *tfoX* genotype. We found that the natural transformation frequencies of *G. parasuis* with different *tfoX* variants were significantly different. The SH0165 *tfoX* groups were not naturally transformable, while SC1401 *tfoX* group exhibited varying degrees of natural transformability (Supplementary Table S3). Therefore, we concluded that *tfoX* is a core regulatory gene for the natural transformation of *G. parasuis*. The molecular level difference of *tfoX* gene can be used as a molecular indicator of whether *G. parasuis* has natural transformability.

### The *tfoX* genotype of SC1401 is an essential factor for the natural transformation of *G. parasuis*

**Figure 3:**
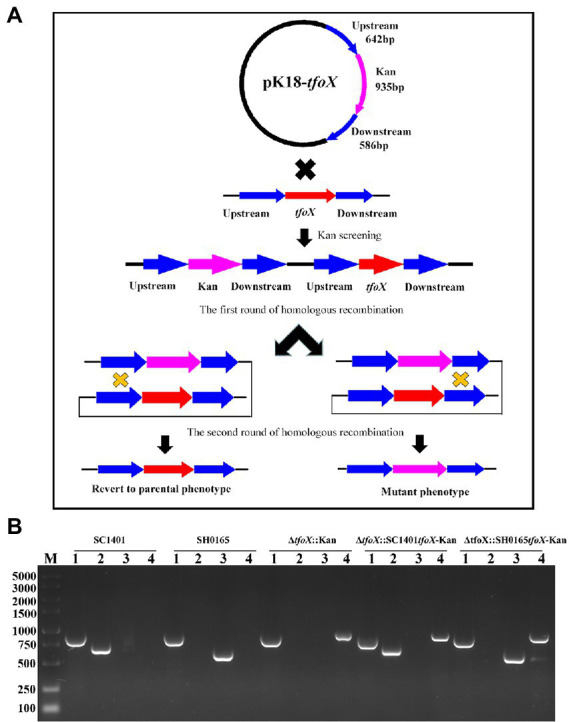
The derivative strains of SC1401, Δ*tfoX*::Kan, Δ*tfoX*::SC1401*tfoX*-Kan, and Δ*tfoX*::SH0165*tfoX*-Kan were constructed by a natural transformation method. **(A)** The schematic diagram of pK18-*tfoX* plasmid and the host genome were recombined in two rounds of homologous recombination to construct *tfoX* gene deletion strains. In the first round of single exchange, the whole plasmid was recombined into the genome. There were both target and resistance genes in the genome of the recipient bacteria; in the second round of recombination, the *tfoX* gene was replaced by Kan. **(B)** PCR verification of SC1401, Δ*tfoX*::Kan, Δ*tfoX*::SC1401*tfoX*-Kan and Δ*tfoX*::SH0165*tfoX*-Kan constructs. Four primer pairs (P17/P18, P9/P10, P19/P20, P21/P22) were used to amplify the *Glaesserella parasuis* 16S rRNA fragment, the *kan* fragment, the SC1401*tfoX* and SH0165*tfoX*, respectively. Lane 1: *G. parasuis* 16S rRNA was amplified from these four strains, respectively; Lane 2: the SC1401*tfoX* gene fragment was amplified from these four strains, respectively; Lane 3: the SH0165*tfoX* gene fragment was amplified from these four strains, respectively; Lane 4: the kanamycin resistance cassette was amplified from these four strains, respectively.

**Figure 4:**
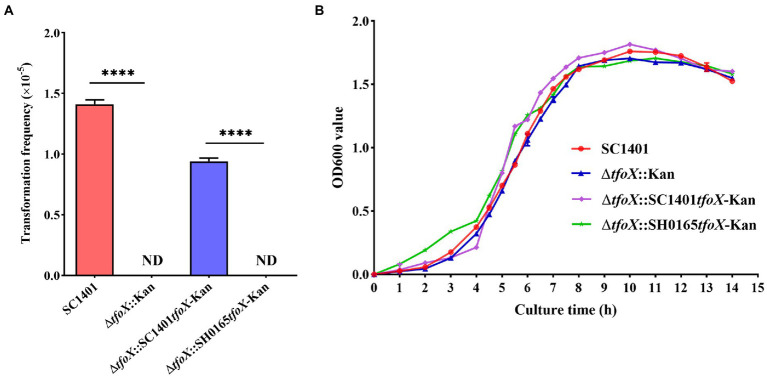
Natural transformation frequency and phenotype of *tfoX* mutant and growth curves of SC1401, Δ*tfoX*::Kan, Δ*tfoX*::SC1401*tfoX*-Kan, and Δ*tfoX*::SH0165*tfoX*-Kan. **(A)** The natural transformation frequency of the wild type and mutant strains. **** value of *p* <0.0001. **(B)** The growth curves of the *Glaesserella parasuis* SC1401 and its derivatives. Bacterial growth was monitored by measuring the optical density at 600 nm at each time point. The results are representative of three independent experiments. Error bars denote the standard deviation.

To further explore the role of *tfoX vis-á-vis* the natural transformation of *G. parasuis*, a *tfoX* gene deletion strain was constructed by naturally transforming with the homologous fragments harboring the suicide plasmid pK18-*tfoX* into SC1401 (Figure 3A). PCR analyses were performed to confirm the proper construction of the Δ*tfoX*::Kan (Figure 3B). As shown in Figure 4A, deletion of *tfoX* leads to a loss of natural competence. However, the Δ*tfoX*::Kan strain did not show significant growth defects *in vitro* when compared with the wildtype SC1401 strains (Figure 4B). These results indicate that *tfoX* is an indispensable factor in the regulation of natural transformation in *G. parasuis*. To further confirm that the SH0165*tfoX* gene leads to the failure of the natural transformation in *G. parasuis*, we constructed the *tfoX* gene differential replacement strains Δ*tfoX*::SH0165*tfoX*-Kan and Δ*tfoX*::SC1401*tfoX*-Kan using SC1401 by the same method as described in Figure 3A above. The results showed that when the *tfoX* gene of SC1401 was replaced with the *tfoX* gene of SH0165, the strain was unable to undergo natural transformation. Similarly, neither of these replacement strains had different growth characteristics *in vitro* (Figures 4A,B). These results confirmed that SC1401*tfoX* is a vital molecular regulatory factor of the natural transformability of *G. parasuis*.

### *tfoX* molecular difference is independent to the expression level

**Figure 5:**
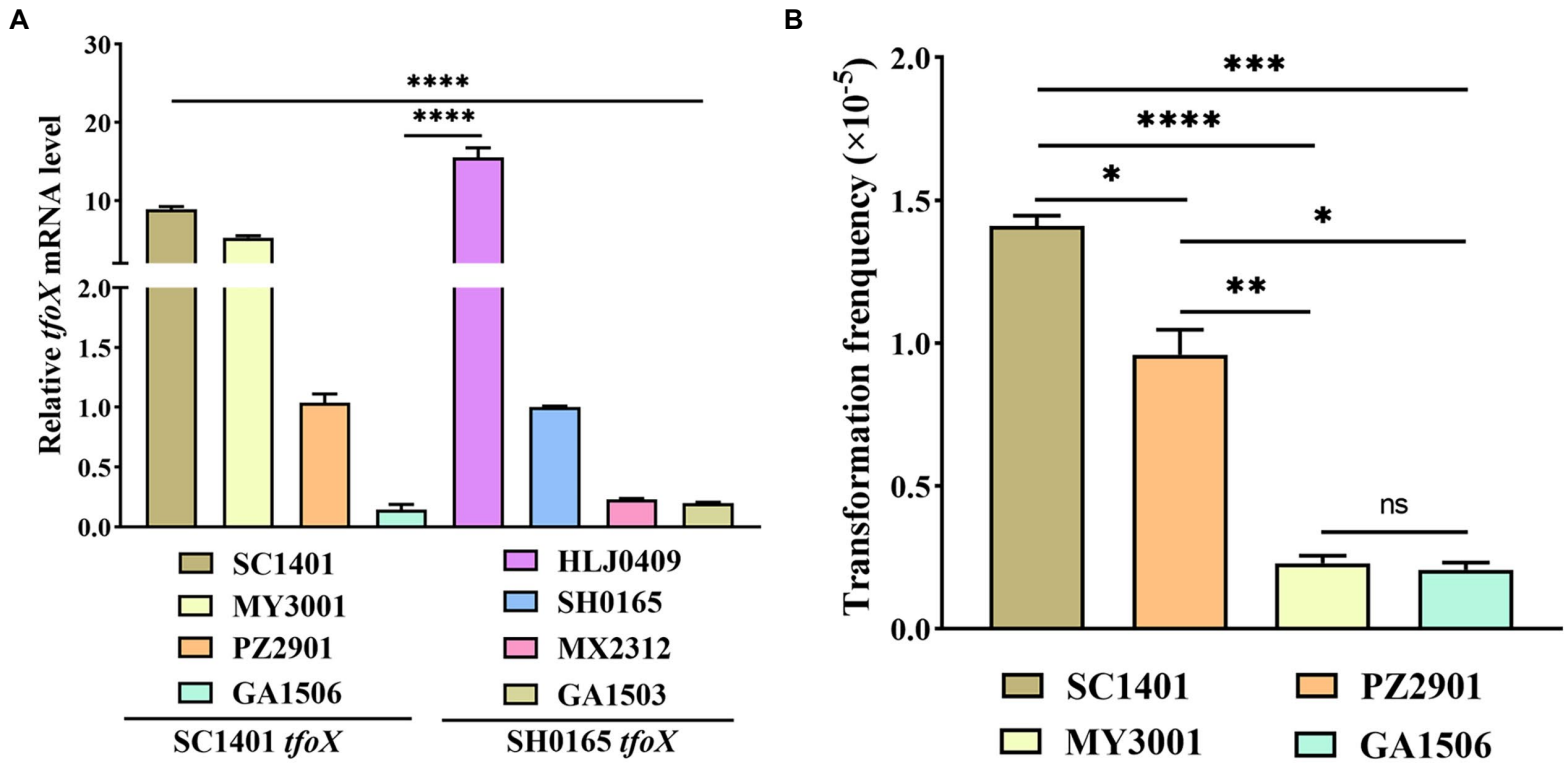
Transcriptional levels of *tfoX* and natural transformation frequency of *Glaesserella parasuis* wild strains. **(A)** Quantitative real-time PCR analysis of the relative transcription of *tfoX* in four each SC1401*tfoX* and SH0165*tfoX* isolates. **(B)** The natural transformation frequency of four SC1401*tfoX* isolates. **** value of p <0.0001; *** value of *p* <0.001; ** value of *p* <0.01; * value of *p* <0.05.

We can confirm that the molecular structure differences of *tfoX* lead to diversity in the natural transformation phenotype, however, the molecular mechanism of natural transformation regulation of *G. parasuis* is unknown. To explore whether *tfoX* variants would result in expression levels of TfoX in SH0165*tfoX* strains *s*ignificantly lower than those of the SC140*tfoX* strains, in addition to SH0165 and SC1401, three of each SH0165*tfoX* and SC140 *tfoX* strains were selected for the analysis of *tfoX* gene transcription levels by fluorescence quantitative PCR. The results showed that not all SC1401 *tfoX* strains have higher *tfoX* gene transcription levels than that of SH0165 *tfoX* strains. Some have lower *tfoX* gene transcription levels (Figure 5A), indicating that such *tfoX* variants are independent of the expression level. Moreover, our results suggested that in different SC1401 *tfoX* strains, the expression level of *tfoX* was not proportional to the natural transformation frequency. Some strains had a high expression level of *tfoX*, but the natural transformation level was low (Figure 5B). This result indicates that the transcription level of *tfoX* alone does not determine the level of natural transformation in different strains. The difference in natural transformation phenotype caused by *tfoX* molecular level difference is not due to the *tfoX* expression level. This result also indicated that *tfoX* is not the only regulatory factor, and many elements also regulate the level of natural transformability.

### Transcriptome sequencing analysis

To further analyze the role of the *tfoX* gene in *G. parasuis*, transcriptomic sequencing was performed on the wildtype strain *G. parasuis* SC1401 and the *tfoX* mutant strain *G. parasuis* SC1401∆*tfoX*::Kan. Three biological replicates were performed. The Total Mapped Reads were all greater than 98%, and Multiple Mapped Reads were all lower than 5% (Supplementary Table S4), indicating that the samples were not contaminated and that the RNA-Seq quality was acceptable. The squared Pearson correlation coefficient (*R*^2^) between the replicate samples was always greater than 0.95 (Supplementary Figure S1), which assured the reliability of the test experiment and the rationality of sample selection. A total of 12 genes were selected for qRT-PCR to test the expression of these genes to validate the reproducibility and repeatability of DEGs identified from transcriptome sequencings (Supplementary Figure S2). The fold changes calculated by qRT-PCR were generally consistent with the trend of the RNA-Seq results, which confirmed the reliability of the RNA-Seq results.

**Figure 6:**
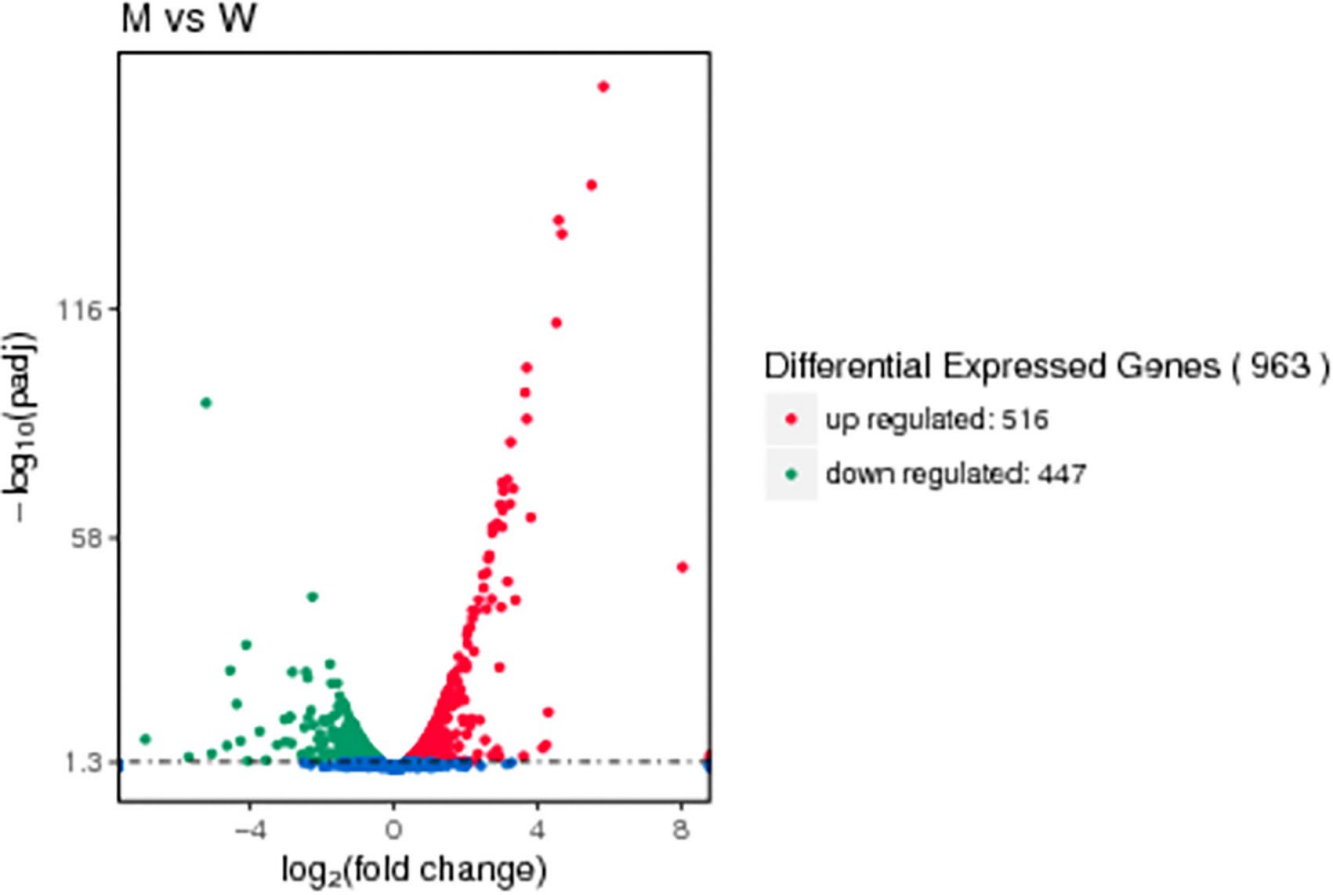
Volcano map of DEGs. Genes with significant differential expression are represented by red dots (upregulated) and green dots (down-regulated), while blue dots represent genes with no significant differences in expression. The abscissa represents the quantitative change in gene expression in the different samples. The ordinate represents the statistical significance of the change in the gene expression levels.

A differential gene expression analysis was performed on the readcount data obtained from the gene expression level analysis to determine the changes in the gene expression profile after deletion of the *tfoX* gene. As shown in the volcano plots (Figure 6), 963 genes were differentially expressed, of which 516 were upregulated and 447 were down-regulated. Supplementary Table S5 shows the readcount of candidate competence genes homologous to that of *H. influenzae* of SC1401 and SC1401∆tfoX::Kan. As listed in Supplementary Table S6, an analysis of the differentially expressed genes (DEGs) revealed 8 competence genes. It is worth noting that almost all were significantly downregulated among the 8 genes except the upregulated gene *ssb*. The 6 downregulated genes, which play crucial roles in DNA uptake, *pilA*, *pilB*, *A4U84_02730*, *pilC*, *comEA*, and *pilF* in SC1401Δ*tfoX* were nearly 7.4-, 4.3-, 3,9-, 2.1-, 2.8-, and 1.4-fold lower than that of in SC1401, respectively. The *comM* gene, which is involved in the transformation process, was downregulated 5.3-fold. Only one gene, *ssb*, which encodes a single-stranded DNA-binding protein that protects DNA from degradation, was slightly upregulated. These competence genes play an essential role in the bacterial uptake of foreign DNA and integration into its genome, suggesting that TfoX may regulate the natural transformability of *G. parasuis* by directly or indirectly activating the expression of these competence genes.

**Figure 7:**
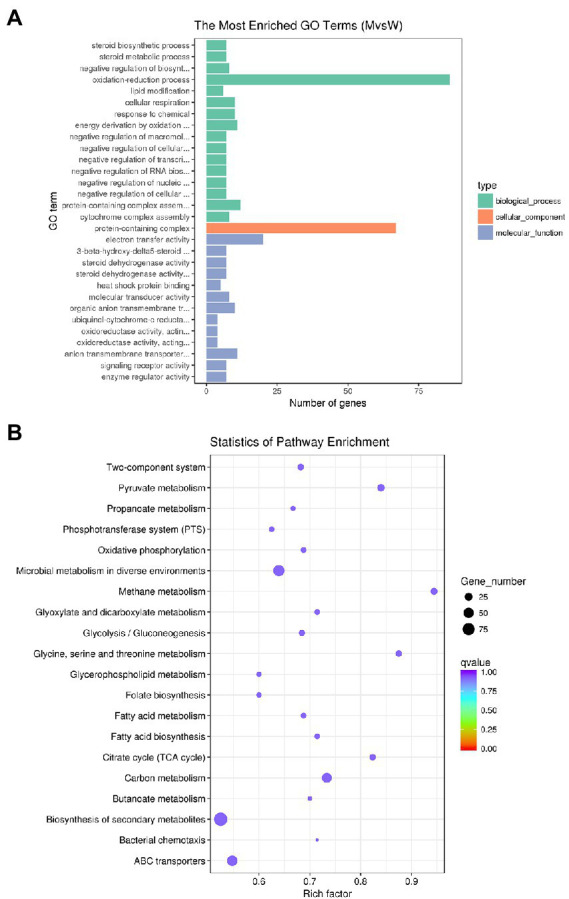
GO and KEGG analysis of DEGs. **(A)** GO enrichment histogram of DEGs. The ordinate is the enriched GO term, and the abscissa is the number of differential genes in this term. Different colors are used to distinguish biological processes, cellular components, and molecular functions. **(B)** Rich distribution points of DEGs in KEGG pathway. The vertical axis represents the pathway name, the horizontal axis represents the enrichment coefficient, the size of the dots represents the number of differentially expressed genes in the pathway, and the dot color corresponds to different qvalue ranges.

Gene ontology (GO) is an international standard classification system of gene functions, which aims to establish linguistic standards for defining and describing the functions of genes and proteins applicable to all species. GO is divided into molecular function, biological process, and cellular components. We analyzed the distribution of DEGs in GO terms to clarify the expression of sample differences in gene function. The 30 GO terms with the most significant enrichment of DEGs are shown in Figure 7A. The most dominant subcategories were oxidation–reduction process, electron transfer activity, protein-containing complexes, energy derivation by oxidation of organic compounds, and anion transmembrane transporter activity. In organisms, different genes coordinate to perform their biological functions, and significant enrichment of pathways can determine the main biochemical metabolic and signal transduction pathways involved by DEGs. The Kyoto Encyclopedia of Genes and Genomes (KEGG) is a database of systematic analysis of gene functions and their pathway networks. A KEGG analysis showed that DEGs were significantly enriched for pathways involved in carbon metabolism, methane metabolism, pyruvate metabolism, and microbial metabolism in diverse environments (Figure 7B).

### Comparative proteomic analysis

**Figure 8:**
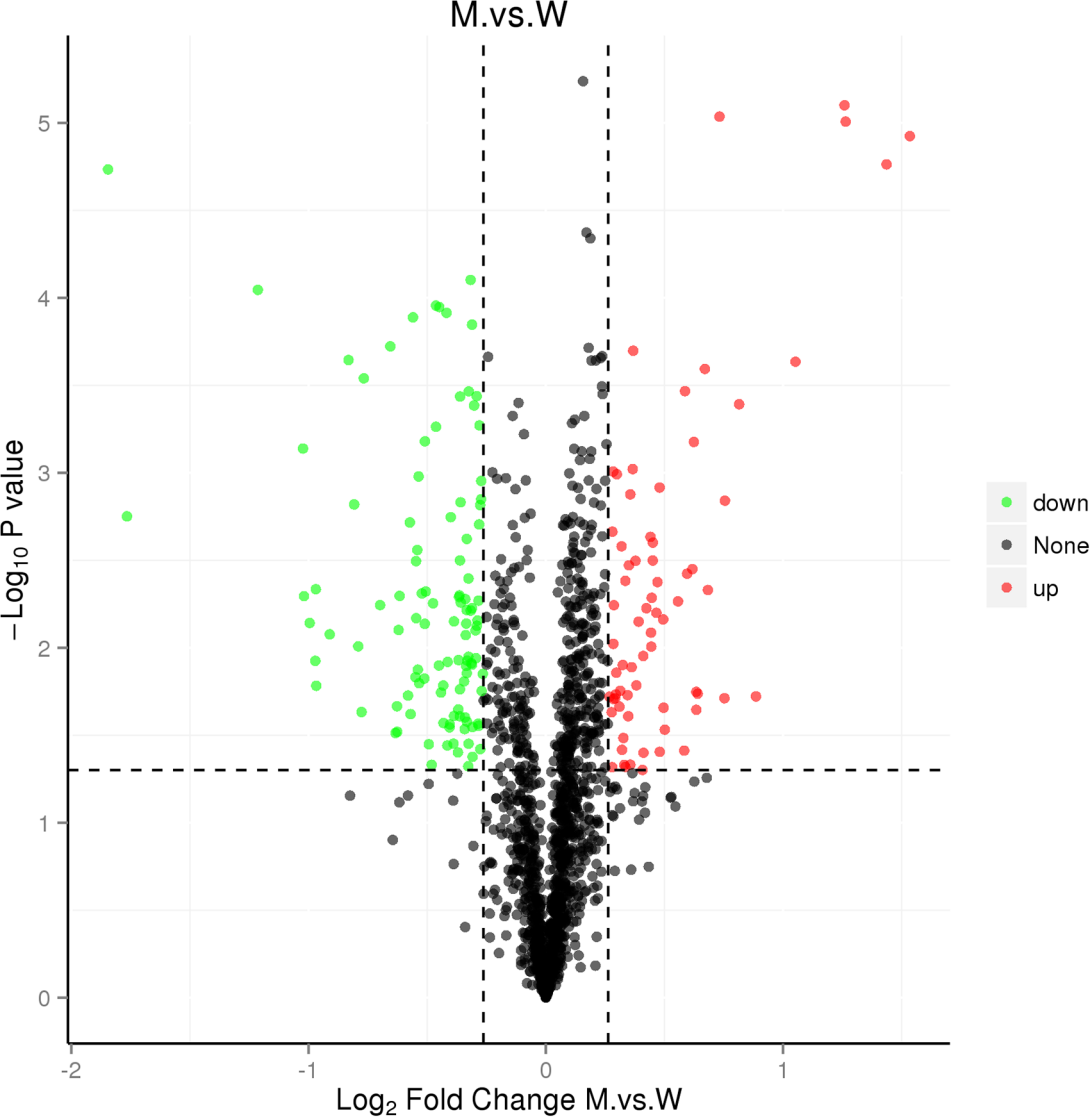
Volcano map of DEPs. The horizontal axis represents the fold change (log2 value) of the DEPs, the vertical axis represents the value of p (−log10 value), black represents proteins with no significant differences, red represents upregulated proteins, and the green represents downregulated proteins.

We further performed a comparative proteomic analysis on the SC1401 and ∆*tfoX*::Kan. As shown in Figure 8, a total of 180 differentially expressed proteins (DEPs) were identified, of which 70 were upregulated (FC ≥ 1.2, *p*-value ≤ 0.05) and 110 were downregulated (FC ≤ 0.83, *p*-value ≤ 0.05).

**Figure 9:**
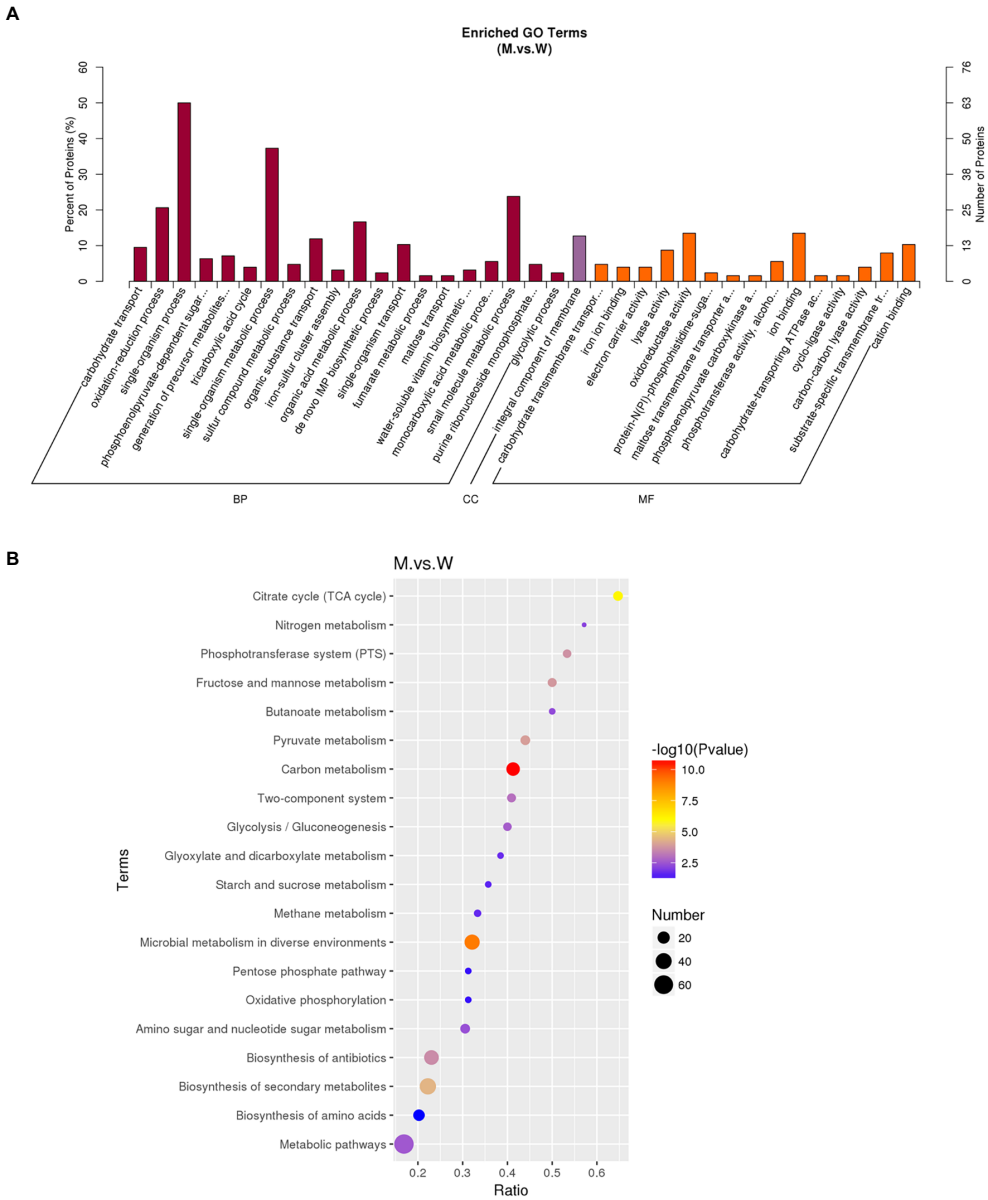
GO and KEGG analysis of DEGs. **(A)** GO enrichment histogram of DEPs. The abscissa is the enriched GO term, and the ordinate is the number of DEPs in this term. The percentage on the ordinate represents the ratio of the number of differential proteins associated with this GO to the number of differential proteins annotated by GO. **(B)** KEGG enrichment bubble diagram of DEPs. The horizontal axis is the ratio of the number of DEPs in the corresponding pathway to the number of total proteins identified. The vertical axis represents the name of the pathway. The color of the point represents the value of p of the hypergeometric test; The size of the dots represents the number of differential proteins in the corresponding pathway.

The DEPs were analyzed by GO enrichment and KEGG enrichment. As shown in Figure 9A, all DEPs were grouped into three GO categories (biological process, cellular component, and molecular function). The GO items enriched by DEPs mainly include single-organism, small molecule metabolic and oxidation–reduction processes in the biological pathways. The GO entries are mainly oxido-reductase activity, ion binding, and cation binding in the molecular function. The GO entries are mainly integral components of the plasma membrane in the cellular component. As shown in Figure 9B, KEGG analysis showed that the main enrichment pathways of DEPs were carbon metabolism, microbial metabolism in diverse environments, fructose and mannose metabolism, citrate cycle, pyruvate, and phosphotransferase system.

## Discussion

Since the first discovery of natural transformation in *Streptococcus pneumoniae* in 1928, many other bacteria have been found to be naturally transformable (Griffith, 1928; Seitz and Blokesch, 2013; Huang et al., 2021). So far, at least 83 species of bacteria have been reported to be capable of natural transformation. However, not all isolates of the transformable species can transform (Evans and Rozen, 2013; Zhang et al., 2021). We found that more than half of *G. parasuis* in laboratory conditions were non-transformable. This result suggests that *G. parasuis*, like *H. influenzae* and *S. pneumoniae*, has a wide range of phenotypic differences related to transformation (Maughan and Redfield, 2009; Evans and Rozen, 2013). It is worth mentioning that the induction conditions are a critical factor for the development of natural transformability of bacteria; for example, high transformability in *H. influenzae* requires induction in starvation M-IV medium. Although the induction conditions for the natural transformation of *G. parasuis* are unknown, it should be emphasized that the environmental conditions in which we showed that some *G. parasuis* isolates were non-transformable were all under laboratory conditions. These strains may develop natural transformability under other specific growth conditions in the field. It was previously suggested that the homologous recombination efficiency of high virulence strains was very low when constructing gene deletion strains by the natural transformation method. It has been speculated that the highly virulent strains have a more restrictive modification system. The foreign DNA was mostly sheared rather than recombined with the target gene to integrate it into the bacterial genome (Zhang et al., 2019). Serotypes have been used as virulence markers in *G. parasuis* (Yu et al., 2014). We found that the percentage of transformable isolates in a group of serotypes that represent high virulence was very low. This result suggests that the natural transformability of bacteria may be related to their virulence. Considering the limited number of experimental strains, further studies are needed to explore the deeper relationship between bacterial virulence and natural transformation.

The natural transformation process occurs due to the precise regulation of many genes, including the core regulatory factors *tfoX* (*sxy*), *crp*, and *cyaA* (Antonova and Hammer, 2015; Simpson et al., 2019). The systematic regulation of the natural transformability of different bacteria, especially gram-negative bacteria, is inconsistent, making it more challenging to study the mechanism of natural transformation (Stutzmann and Blokesch, 2020). With the recent emergence of advanced genomics and transcriptomic sequencing, researchers have gradually discovered the internal mechanism of differential natural transformation phenotypes in several strains (Cohen et al., 2021). For instance, Durieux et al. used genome-wide association studies and found that the loss of the transformation of *Legionella pneumophila* was associated with the loss of a conjugative plasmid. (Attaiech et al., 2016). SC1401*tfoX* and SH0165*tfoX* have significant molecular level differences, only 74 and 73% homology of the DNA and amino acid sequences, respectively. Such molecular diversity of *tfoX* has not been reported in bacteria such as *H. influenzae* or *E. coli*. We speculated that the differential phenotypes of natural transformation of *G. parasuis* were caused by molecular level differences in *tfoX*, as SH0165 failed to transform under laboratory culture conditions.

High resolution melting (HRM) technology plays a vital role in genotyping (Li et al., 2019; Soejima and Koda, 2021). Through HRM and sequencing analyses, we determined that all *tfoX* genes can be divided into two types. Further study showed that all of SH0165*tfoX* laboratory strains were non-transformable, while SC1401*tfoX* strains had different natural transformability levels. These results indicate that *G. parasuis* may have a unique natural transformation regulatory system, and *tfoX* can be used as a molecular marker to distinguish whether a particular *G. parasuis* isolate will be naturally transformable. In addition, the previously reported *G. parasuis* molecular classification method may be somewhat complex and not applicable to specific strains. Meanwhile, all *G. parasuis* strains could be divided into two types using *tfoX* molecular level differences, namely, SC1401*tfoX* and SH0165*tfoX*, respectively representing strains with or without natural transformability. Such results provide a new perspective for *G. parasuis* molecular typing.

The complete loss of the natural transformability of Δ*tfoX*::Kan and Δ*tfoX*::SH0165*tfoX*-Kan indicated that SC1401TfoX is an indispensable factor in the regulation of natural transformation. This result showed that SH0165 failed to undergo natural transformation which was mediated independently by *tfoX*. Previous studies on the natural transformation of *H. influenzae* showed that the transcription and translation efficiency of a *sxy* gene mutation strain was greatly improved. However, our experiments have shown that the *tfoX* variants did not affect expression levels. The diversity in the natural transformability of *G. parasuis* is due to the structure of *tfoX per se*, which leads *tfoX* genes to perform different functions of activating or inhibiting downstream genes, or the activation function of SH0165*tfoX* is affected by its structure. The natural competence of *S. pneumoniae* is regulated by the ComD protein, whose histidine kinase activity is regulated by the competence stimulating peptide (CSP; Yang et al., 2017). The majority of *S. pneumoniae* strains produce one of two variants of CSP, namely CSP1 or CSP2, and the cognate receptors ComD1 and ComD2, respectively (Yang and Tal-Gan, 2019). In *E. coli*, TfoX could directly interact with cAMP Receptor Protein (CRP; Sondberg et al., 2019). However, CRP is conserved in *G. parasuis*. Identifying the proteins that interact with TfoX will be a key step in understanding the molecular mechanism of how the two TfoX proteins regulate natural transformation. In addition, the expression level of *tfoX* was not positively correlated with the natural transformation frequency in SC1401*tfoX* strains. The reason is unknown. It could be that the regulation of natural transformation is a complex network, and different strains could be affected by many other factors.

Multiple competence genes were significantly down-regulated in *tfoX* mutant strains compared to the wildtype, including *pilA, comM*, *pilB*, *A4U84_02730*, *comEA*, *pilC*, and *pilF.* Proteins encoded by those genes play an essential role in the uptake and transport of foreign DNA (Nero et al., 2018; Dalia and Dalia, 2019). Our results indicated that SC1401*tfoX* can activate transcription of downstream genes associated with natural transformation in *G. parasuis*. In various bacteria, TfoX synergistically binds the cAMP-CRP complex to the CRP binding site in the promoter region of the competence gene, thereby initiating transcription (Jaskolska and Gerdes, 2015). CRP is a cyclic adenosine monophosphate (cAMP) receptor protein widely existing in the *γ-proteobacteria* (Redfield et al., 2005; Cameron and Redfield, 2008). In *G. parasuis*, the interaction between TfoX and CRP is unknown. However, we found that CRP proved to be an indispensable factor in regulating natural transformation in *G. parasuis* SC1401 in an independent experiment. When the *crp* gene was deleted, natural transformability was lost. Further, if the adenylate cyclase coding gene *cyaA* was deleted, the natural transformation level of *G. parasuis* decreased to a very low level (data not shown). These results suggest that the cAMP-CRP complex also plays a vital role in *G. parasuis* natural transformation. Further studies to dissect the binding affinity of the two TfoX proteins to the cAMP-CRP complex is needed.

A transcriptomic analysis showed that 936 genes were differentially expressed and a proteomics analysis showed that 180 proteins were differentially expressed. These results indicated that TfoX had an overall regulatory effect in the bacterium. Differentially expressed genes and protein accumulation pathways mostly revolve around sugar metabolism. As a fundamental element of microbial growth, the carbon source participates in various metabolic processes and regulatory pathways (Macfadyen and Redfield, 1996). The effect of carbon sources on natural transformation has been reported in many other bacteria. For example, transformable states of *H. influenzae* are affected by the PTS carbon source coupled cAMP (Macfadyen et al., 1996; Macfadyen and Redfield, 1996; Sinha et al., 2013). Carbon source metabolism raises the intracellular cAMP content *via* PTS, and then influences the expression of downstream receptor genes initiated by the transcriptional regulator cAMP-CRP (Guo et al., 2015). Regulatory effects of TfoX on carbon metabolism has not been reported in other bacteria. Still in the triple regulatory network of natural transformation in *V. cholerae*, *tfoX* transcription is induced by the chitin pathway, and TfoX expression in *E. coli* is directly regulated by the cAMP-CRP complex (Sondberg et al., 2019; Stutzmann and Blokesch, 2020). Whether there is a bidirectional regulation mechanism between TfoX and carbon source metabolic pathways or cAMP must be determined in future studies.

An in-depth analysis of the biological significance of natural transformation in bacteria can provide a new perspectives for studies of bacterial virulence changes, the horizontal spread of antibiotic resistance cassettes and genetic evolution. *G. parasuis* is a naturally transformable bacterium, and has been mainly used currently to create a set of gene manipulation techniques and to construct gene deletion strains for functional studies (Zhang et al., 2015, 2019; Jiang et al., 2020). However, the natural transformation mechanism in *G. parasuis* is unknown. In this study, we focused on the genetic heterogenicity of *tfoX* as represented by SC1401*tfoX* and SH0165*tfoX*, and discovered that it is an essential determinant for the natural transformation of *G. parasuis*. We have also preliminarily explored *tfoX* regulatory pathways. Future studies to understand the structural differences of the two TfoX variants and their regulatory functions *via* á vis the natural transformation phenotypes of *G. parasuis* are warranted.

## Data availability statement

The datasets presented in this study can be found in online repositories. The names of the repository/repositories and accession number(s) can be found at: https://www.ncbi.nlm.nih.gov/, PRJNA813391; https://www.iprox.cn/, IPX0004196000.

## Conflict of interest

The authors declare that the research was conducted in the absence of any commercial or financial relationships that could be construed as a potential conflict of interest.

## Publisher’s note

All claims expressed in this article are solely those of the authors and do not necessarily represent those of their affiliated organizations, or those of the publisher, the editors and the reviewers. Any product that may be evaluated in this article, or claim that may be made by its manufacturer, is not guaranteed or endorsed by the publisher.

## Author contributions

XT, ZY, and KD: designed the experiments. Y-FC, SC, XHu, RW, QZ, YH, SD, YL, XW, and XHa: performed the experiments with assistance from GL, XT, YZ, and BH. XT, ZY, YW, and KD: analyzed the data and wrote the paper. All the authors reviewed and approved the final manuscript.

## Funding

This work was funded by the Chengdu Science and Technology Bureau key R&D Support Plan (2022-YF05-00817-SN).

## Footnotes

^1^ https://itol.embl.de/ (Accessed July 16, 2022)

^2^ https://www.ncbi.nlm.nih.gov/nuccore/CP015099.1 (Accessed July 16, 2022)

^3^ https://www.ncbi.nlm.nih.gov/bioproject/PRJNA317522 (Accessed July 7, 2022)
