# Supplementary material for: The molecular diversity of transcriptional factor TfoX is a determinant in natural transformation in Glaesserella parasuis
Source: Front Microbiol. 2022 Jul 29;13:948633. doi: 10.3389/fmicb.2022.948633 (PMC9372613; doi:10.3389/fmicb.2022.948633)
Supplement: Supplementary file 1 [file Data_Sheet_1.pdf]

Supplementary Material

Supplementary Tables

Table S1. Strains and plasmids were used in this study.

Strains or Plasmids	Relevant characteristic(s)	Source
G. parasuis		
SC1401	Serotype 11 clinical isolate, highly transformable strain	Laboratory collection
SH0165	Serotype 5 clinical isolate, non-transformable strain	Laboratory collection
GA1503	Serotype 4 clinical isolate, non-transformable strain	Laboratory collection
MX2312	Serotype 5 clinical isolate, non-transformable strain	Laboratory collection
HLJ0409	Serotype 14 clinical isolate, non-transformable strain	Laboratory collection
GA1506	Serotype 4 clinical isolate, transformable strain	Laboratory collection
MY1902	Serotype 5 clinical isolate, transformable strain	Laboratory collection
MY3001	Serotype 7 clinical isolate, transformable strain	Laboratory collection
SC1401Δ*htrA*::Kan	SC1401 derivative, *htrA* deletion, Kan^R	Laboratory collection
SC1401Δ*cdt*::Erm	SC1401 derivative, *cdt* deletion, Erm^R	Laboratory collection
Δ*tfoX*::Kan	SC1401 derivative, *tfoX* deletion, Kan^R	This study
Δ*tfoX*::SC1401*tfoX*-Kan	SC1401 derivative, *tfoX* was replaced by fusion fragment SC1401*tfoX*-Kan, Kan^R	This study
Δ*tfoX*::SH0165*tfoX*-Kan	SC1401 derivative, *tfoX* was replaced by fusion fragment SH0165*tfoX*-Kan, Kan^R	This study
E. coli		
DH5α	Standard *E. coli* cloning strain	Biomed
**Plasmids**		
pK18mobsacB	Suicide and narrow-broad-host vector, Kan^R	Laboratory collection
pKD4	Kanamycin resistance cassette-carrying vector, Kan^R	Laboratory collection

pK18-*tfoX*	A 2163-bp fragment containing the kanamycin resistance cassette, upstream and downstream sequence of *tfoX* in pK18mobSacB, Kan^R	This study
pK18-SC1401*tfoX*-Kan	A 2805-bp fragment containing the kanamycin resistance cassette, upstream and downstream sequence of *tfoX* gene and SC1401*tfoX* gen in pK18mobSacB, Kan^R	This study
pK18-SH0165*tfoX*-Kan	A 2802-bp fragment containing the kanamycin resistance cassette, upstream and downstream sequence of *tfoX* and SH0165*tfoX* gen in pK18mobSacB, Kan^R	This study

Kan^R kanamycin resistance, Erm^R erythromycin resistance.

Table S2. Primers used in this study.

Primers	Primer sequences (5′→3′)	products (bp)
**Conventional primers**		
P1(*tfoX*-HRM-F)	ATGAAATACATTGATGTAAAAACACAA	108
P2(*tfoX*-HRM-R)	ATCTTTCATTATTCCATAGTAGGAAA	
P3(*tfoX*-seq-F)	ATGAAATACATTGATGTAAAAACA	1004
P4(*tfoX*-seq-R)	AAATCACGTTTTTCAGCAGAA	
P5(*tfoX*L-F)	ctatgacatgattacgaattcTGATTGGCTGAGCAAAGCG	642
P6(*tfoX*L-R)	gcagggcttcccaaccttacAAAAAAAACCTCGTTTGCGAT	
P7(*tfoX*R-F)	ggggttcgaaatgaccgaccCACCTGCTTCTTATGTTGGA	586
P8(*tfoX*R-R)	caggtcgactctagaggatccCTGTTGGGTAAGTGTCGTTT	
P9(Kan-F)	GTAAGGTTGGGAAGCCCTGC	935
P10(Kan-R)	GGTCGGTCA TTTCGAACCCC	
P11(1401*tfoX*-F)	tgtatcgcaaacgaggttttttttATGAAATACATTGATGTAAAAACAC	642
P12(1401*tfoX*-R)	tgtgttttatatttttctcgttcatTTATTTAACATTGAAGCGCTTAC	
P13(0165*tfoX*-F)	tgtatcgcaaacgaggttttttttATGAAATACATTGATGCAAAAAC	639
P14(0165*tfoX*-R)	tgtgttttatatttttctcgttcatTTATCTAGTATTAAATCGCTTTCG	
P15 (pK18-F)	CTGGCACGACAGGTTTCC	342
P16(pK18-R)	GCCTCTTCGCTA TTACGC	
P17(HPS-F)	GTGATGAGGAAGGGTGGTGT	822
P18(HPS-R)	GGCTTCGTCACCCTCTGT	
P19(1401-F)	ATGAAATACATTGATGTAAAAACAC	642
P20(1401-R)	TTATTTAACATTGAAGCGCTTAC	
P21(0165-F)	AATCATTGGCGAAACGAAAGC	560

P22(0165-R)	CAGCAGCATACAAGGCATTATC	
**qPCR primers**		
16S-F	TGGTAGTCCACGCTGTAAAC	201
16S-R	AGGATGTCAAGAGTAGGTAAGG	
tfox-SC1401-F	TGTATCCGTACCTTACCTAATATG	179
tfox-SC1401-R	CGATTAATTGCTCCATAAAGACG	
tfox-SH0165-F	ATTTAGTAGATAGAGGCGAGATTGA	180
tfox-SH0165-R	CAGCAGCATACAAGGCATTATC	
A4U84_02360-F	ACACAAGCCAAGTTGCCCAAAATC	133
A4U84_02360-R	GCCATAACCCGCACCAAGAGAAG	
A4U84_02570-F	GTTGGCTGTTAGTGGGCAATTAGC	120
A4U84_02570-R	TGCCCCTCATCAACAGAAACAGTG	
A4U84_02575-F	CATTGCTGGCGGCTCAAGAGG	92
A4U84_02575-R	GGCGAACCCCATCAATCACCTG	
A4U84_10385-F	CTCAATGCTCAGTGGCGTGGATAG	83
A4U84_10385-R	GACCAATAGCAAGAGTGCCGACAG	
A4U84_08565-F	GTTCAACGGCACAGCAAGG	145
A4U84_08565-R	TCACCCATTCCCATCATCACC	
A4U84_00055-F	GTTAGACAGCCGTAGCAGTGG	96
A4U84_00055-R	TAGCCGCCTTGATTGTAGTTGG	
A4U84_06330-F	CACACCTGAGCCACAGCCTAATTC	150
A4U84_06330-R	TCATCGCCACAACCGTATCAATCG	
A4U84_06335-F	GCTATGCGGTGGACGAGTTAAGTG	103
A4U84_06335-R	GCACGGCAGGCACAGAGTTC	
A4U84_06325-F	TGGACATCGGCGTAATGCTACTTG	93
A4U84_06325-R	TCGCTTGTTGCTTCGCTCTATCTG	
A4U84_08525-F	GCATACCAACGACGCACTCTC	144
A4U84_08525-R	TCACAGCCTACTCCACGACAC	
A4U84_07375-F	AGGCTCAGGCAATTGTGGAA	95
A4U84_07375-R	AAGTGTCGCTTTCCCGATACC	
A4U84_06900-F	GCATTATCAAGGAACGCACAATCG	117
A4U84_06900-R	GGCAGTAACGGCACTTCAATCG	

Lowercase letters are homologous recombination fragments required for in-fusion cloning to the adjoining segments or vectors.

Table S3. 99 GPS strains naturally transformation phenotype and *tfoX* type.

Sequence number	strain name	serotype	source	*tfoX* type	Natural transformability	Natural transformation frequency
1	SC1401	11	Sichuan, China	SC1401	transformable	1.47×10^{-4}
2	MX1707	1	Sichuan, China	SC1401	transformable	0.19×10^{-7}
3	MX2101	1	Sichuan, China	SC1401	transformable	1.32×10^{-5}
4	HY12001	2	Sichuan, China	SC1401	transformable	1.12×10^{-5}
5	MX1704	2	Sichuan, China	SC1401	transformable	0.86×10^{-4}
6	YC0201	2	Sichuan, China	SC1401	transformable	1.23×10^{-5}
7	SN0402	2	Sichuan, China	SC1401	transformable	0.98×10^{-7}
8	SN0403	2	Sichuan, China	SC1401	transformable	1.14×10^{-5}
9	HB0503	2	Hebei, China	SC1401	transformable	0.21×10^{-7}
10	SN0401	2	Sichuan, China	SC1401	transformable	0.86×10^{-6}
11	MX1206	4	Sichuan, China	SC1401	transformable	0.71×10^{-5}
12	GA1506	4	Sichuan, China	SC1401	transformable	0.16×10^{-7}
13	XJ1207	4	Sichuan, China	SC1401	transformable	0.87×10^{-5}
14	MS0105	4	Sichuan, China	SC1401	transformable	1.01×10^{-4}
15	PZ2904	5	Sichuan, China	SC1401	transformable	0.53×10^{-5}
16	WJ1703	5	Sichuan, China	SC1401	transformable	0.47×10^{-5}
17	YA1801	5	Sichuan, China	SC1401	transformable	0.44×10^{-5}
18	PZ2901	5	Sichuan, China	SC1401	transformable	1.02×10^{-5}
19	PZ2902	5	Sichuan, China	SC1401	transformable	0.11×10^{-6}
20	QL3001	5	Sichuan, China	SC1401	transformable	0.92×10^{-6}
21	MY1902	5	Sichuan, China	SC1401	transformable	0.54×10^{-5}
22	QL2801	5	Sichuan, China	SC1401	transformable	0.17×10^{-6}
23	MX2103	7	Sichuan, China	SC1401	transformable	0.24×10^{-6}
24	MY3001	7	Sichuan, China	SC1401	transformable	0.86×10^{-4}
25	MS1902	7	Sichuan, China	SC1401	transformable	0.18×10^{-6}
26	LS1908	7	Sichuan, China	SC1401	transformable	0.25×10^{-6}
27	XJ2101	7	Sichuan, China	SC1401	transformable	0.32×10^{-6}
28	SP0601	7	Sichuan, China	SC1401	transformable	0.33×10^{-6}

29	XJ2102	7	Sichuan, China	SC1401	transformable	0.19×10^{-6}
30	YA0801	11	Sichuan, China	SC1401	transformable	0.45×10^{-6}
31	XC1905	12	Sichuan, China	SC1401	transformable	0.48×10^{-6}
32	LZ2901	12	Sichuan, China	SC1401	transformable	0.26×10^{-7}
33	MS1001	13	Sichuan, China	SC1401	transformable	0.65×10^{-6}
34	XJ3105	13	Sichuan, China	SC1401	transformable	0.23×10^{-7}
35	XJ3103	13	Sichuan, China	SC1401	transformable	0.19×10^{-7}
36	S1	1	Sichuan, China	SC1401	transformable	0.48×10^{-5}
37	S2	2	Sichuan, China	SC1401	transformable	1.01×10^{-5}
38	S3	3	Sichuan, China	SC1401	transformable	0.19×10^{-6}
39	S4	4	Sichuan, China	SC1401	transformable	0.18×10^{-6}
40	S6	6	Sichuan, China	SC1401	transformable	0.22×10^{-6}
41	S7	7	Sichuan, China	SC1401	transformable	0.14×10^{-5}
42	S8	8	Sichuan, China	SC1401	transformable	0.21×10^{-5}
43	S9	9	Sichuan, China	SC1401	transformable	0.27×10^{-5}
44	S11	5	Sichuan, China	SC1401	transformable	0.23×10^{-7}
45	SH0165	5	Hebei, China	SH0165	non-transformable	0
46	XJ3104	1	Sichuan, China	SH0165	non-transformable	0
47	MS0804	1	Sichuan, China	SH0165	non-transformable	0
48	XC2401	4	Sichuan, China	SH0165	non-transformable	0
49	MX1201	4	Sichuan, China	SH0165	non-transformable	0
50	GA1503	4	Sichuan, China	SH0165	non-transformable	0
51	GA1504	4	Sichuan, China	SH0165	non-transformable	0
52	DY3102	4	Sichuan, China	SH0165	non-transformable	0
53	ZD1001	4	Sichuan, China	SH0165	non-transformable	0
54	MX2104	4	Sichuan, China	SH0165	non-transformable	0
55	CZ1301	4	Sichuan, China	SH0165	non-transformable	0

56	MY1001	4	Sichuan, China	SH0165	non-transformable	0
57	XJ1901	4	Sichuan, China	SH0165	non-transformable	0
58	XJ1902	4	Sichuan, China	SH0165	non-transformable	0
59	GA1505	4	Sichuan, China	SH0165	non-transformable	0
60	MX1705	4	Sichuan, China	SH0165	non-transformable	0
61	ZD1002	4	Sichuan, China	SH0165	non-transformable	0
62	MX2102	4	Sichuan, China	SH0165	non-transformable	0
63	YB2002	5	Sichuan, China	SH0165	non-transformable	0
64	WJ1702	5	Sichuan, China	SH0165	non-transformable	0
65	HB0502	5	Hebei, China	SH0165	non-transformable	0
66	HB0501	5	Hebei, China	SH0165	non-transformable	0
67	QL1201	5	Sichuan, China	SH0165	non-transformable	0
68	HB0505	5	Hebei, China	SH0165	non-transformable	0
69	EM1901	5	Sichuan, China	SH0165	non-transformable	0
70	MX2308	5	Sichuan, China	SH0165	non-transformable	0
71	MX2312	5	Sichuan, China	SH0165	non-transformable	0
72	MS1503	5	Sichuan, China	SH0165	non-transformable	0
73	YB2001	5	Sichuan, China	SH0165	non-transformable	0
74	MX1708	5	Sichuan, China	SH0165	non-transformable	0
75	XC2602	5	Sichuan, China	SH0165	non-transformable	0
76	HLJ0403	5	Heilongjiang， China	SH0165	non-transformable	0

77	XJ0401	5	Sichuan, China	SH0165	non-transformable	0
78	XJ0902	5	Sichuan, China	SH0165	non-transformable	0
79	MC1202	5	Sichuan, China	SH0165	non-transformable	0
80	GA1501	5	Sichuan, China	SH0165	non-transformable	0
81	MS1502	5	Sichuan, China	SH0165	non-transformable	0
82	XC2603	5	Sichuan, China	SH0165	non-transformable	0
83	HLJ0402	5	Heilongjiang，China	SH0165	non-transformable	0
84	JJ0801	5	Sichuan, China	SH0165	non-transformable	0
85	YC2401	5	Sichuan, China	SH0165	non-transformable	0
86	MX1701	5	Sichuan, China	SH0165	non-transformable	0
87	HPS0109	12	Sichuan, China	SH0165	non-transformable	0
88	HPSH4	12	Sichuan, China	SH0165	non-transformable	0
89	XJ2103	12	Sichuan, China	SH0165	non-transformable	0
90	HLJ4012	13	Heilongjiang，China	SH0165	non-transformable	0
91	HS145	14	Sichuan, China	SH0165	non-transformable	0
92	HLJ0409	14	Heilongjiang，China	SH0165	non-transformable	0
93	HPS0123	14	Sichuan, China	SH0165	non-transformable	0
94	S5	5	Sichuan, China	SH0165	non-transformable	0
95	S10	10	Sichuan, China	SH0165	non-transformable	0
96	S12	12	Sichuan, China	SH0165	non-transformable	0
97	S13	13	Sichuan, China	SH0165	non-transformable	0

98	S14	14	Sichuan, China	SH0165	non-transformable	0
99	S15	15	Sichuan, China	SH0165	non-transformable	0

Transformation frequencies were determined from the number of antibiotic-resistant cfu mL^{-1} divided by the total cfu mL^{-1} scored on non-selective agar. Each experiment was performed with three biological replicates, and the data in the table are the average of the three replicates.

Table S4. A list of Reads compared with reference genomes.

Sample name	M1	M2	M3	W1	W2	W3
Total reads	12043904	11347334	12734186	19630800	20467008	25807682
Total mapped	11928825 (99.04%)	11231736 (98.98%)	12622241 (99.12%)	19462012 (99.14%)	20100910 (98.21%)	25430418 (98.54%)
Multiple mapped	259735 (2.16%)	236535 (2.08%)	267471 (2.1%)	535662 (2.73%)	637384 (3.11%)	863595 (3.35%)
Uniquely mapped	11669090 (96.89%)	10995201 (96.9%)	12354770 (97.02%)	18926350 (96.41%)	19463526 (95.1%)	24566823 (95.19%)
Read-1	5837415 (48.47%)	5499816 (48.47%)	6180282 (48.53%)	9466769 (48.22%)	9738481 (47.58%)	12292500 (47.63%)
Read-2	5831675 (48.42%)	5495385 (48.43%)	6174488 (48.49%)	9459581 (48.19%)	9725045 (47.52%)	12274323 (47.56%)
Reads map to '+'	5834921 (48.45%)	5497885 (48.45%)	6177713 (48.51%)	9463528 (48.21%)	9732534 (47.55%)	12286989 (47.61%)
Reads map to '-'	5834169 (48.44%)	5497316 (48.45%)	6177057 (48.51%)	9462822 (48.2%)	9730992 (47.54%)	12279834 (47.58%)

Table S5. Readcount of competence genes of SC1401 and SC1401Δ*tfoX*::Kan

**Gene ID**	**Gene**	**readcount**					
		**M1**	**M2**	**M3**	**W1**	**W2**	**W3**
A4U84_02030	*comA*	15	17	14	44	51	61
A4U84_02025	*comB*	6	1	2	11	9	6
A4U84_02020	*comC*	2	0	1	5	7	8
A4U84_02015	*comD*	1	0	4	6	6	6
A4U84_02010	*comE*	89	95	114	160	165	161
A4U84_05740	*comF*	24	25	27	60	49	57
A4U84_07375	*comEA*	170	160	181	787	812	1295
A4U84_08530	*pilA*	18	10	21	198	206	316
A4U84_08525	*pilB*	15	30	19	209	143	205
A4U84_08520	*pilC*	6	16	6	43	31	48

A4U84_08515	*pilD*	5	5	8	25	18	13
A4U84_02240	*comN*	6	6	11	26	21	28
A4U84_02230	*comP*	8	7	2	23	27	16
A4U84_02225	*comQ*	24	21	18	38	31	41
A4U84_10535	*comEC*	120	108	121	280	203	232
A4U84_02735	*pilF*	136	137	145	388	451	343
A4U84_02730	*HI0365*	488	506	596	4016	3335	5179
A4U84_06900	*comM*	99	130	120	1325	1195	1145
A4U84_04945	*dprA*	217	185	182	466	472	558
A4U84_07560	*recA*	3988	3620	4094	6218	6349	6453
A4U84_01895	*ligA*	934	988	1061	2067	2493	2096
A4U84_00055	*ssb*	2854	2744	3141	4071	3581	3352
A4U84_03695	*radC*	45	45	47	108	108	157
A4U84_01305	*murE*	2320	2120	2439	6515	5896	6801
A4U84_08565	*HI1631*	50	46	61	45	34	42

Table S6. Significantly differentially expressed competence genes

Gene ID	gene	Description	Log2(Flod change)
A4U84_08530	*pilA*	prepilin-type N-terminal cleavage	-2.8778
A4U84_06900	*comM*	ATP-dependent protease	-2.3964
A4U84_08525	*pilB*	protein transporter HofB	-2.1114
A4U84_02730	/	ribosomal RNA large subunit methyltransferase RlmN	-1.9743
A4U84_07375	*comEA*	transporter	-1.4875
A4U84_08520	*pilC*	fimbrial protein	-1.0946
A4U84_02735	*pilF*	type IV pilus biogenesis	-0.50509
A4U84_00055	*ssb*	Single-strand DNA-binding protein	0.65714

Supplementary Figures

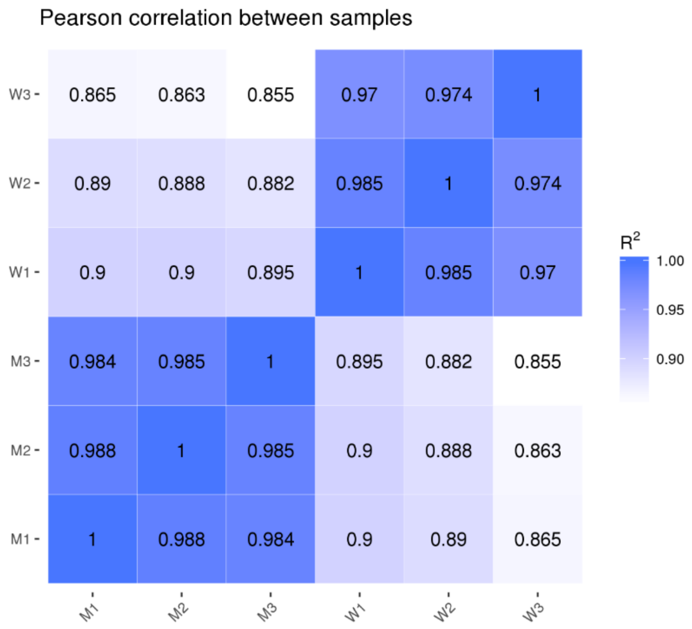

Figure S1. Heat map of the correlation coefficient between samples. M1-M3 represents three biological duplications of deletion strain, W1-W3 represents three biological duplications of wild strain, R^2 represents the square of Pearson correlation coefficient. R^2 ＞0.95 between the three biological replicates, indicating a good correlation.

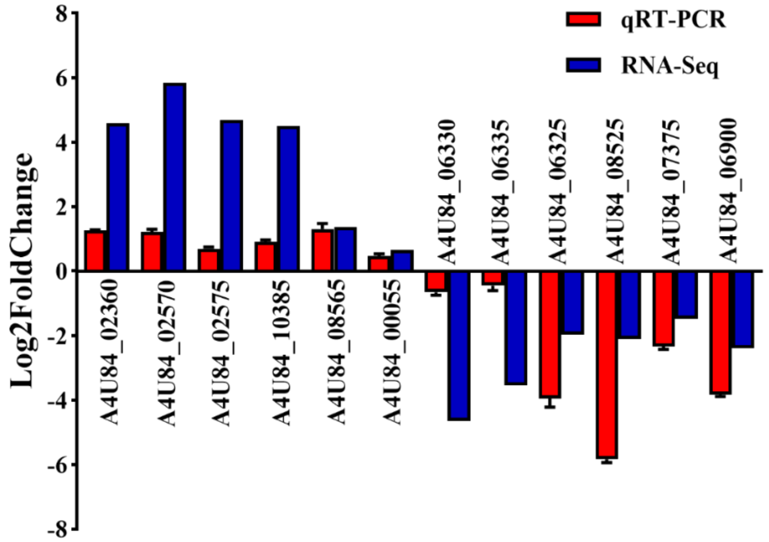

Figure S2. Validation of RNA-Sequencing results by quantitative RT-PCR. Log2 fold change comparison of RNA-Seq (blue bars) and quantitative real-time PCR (red bars) for six differentially expressed genes upregulated at Δ*tfoX*::Kan, six genes downregulated at Δ*tfoX*::Kan. RT-PCR results showed general consistency with RNA-seq data.
